# Antibody-Based Therapy for Enterococcal Catheter-Associated Urinary Tract Infections

**DOI:** 10.1128/mBio.01653-16

**Published:** 2016-10-25

**Authors:** Ana L. Flores-Mireles, Jennifer N. Walker, Aaron Potretzke, Henry L. Schreiber, Jerome S. Pinkner, Tyler M. Bauman, Alyssa M. Park, Alana Desai, Scott J. Hultgren, Michael G. Caparon

**Affiliations:** a Department of Molecular Microbiology and Center for Women’s Infectious Disease Research, Washington University School of Medicine, Saint Louis, Missouri, USA; b Department of Surgery, Washington University School of Medicine, Saint Louis, Missouri, USA

## Abstract

Gram-positive bacteria in the genus *Enterococcus* are a frequent cause of catheter-associated urinary tract infection (CAUTI), a disease whose treatment is increasingly challenged by multiantibiotic-resistant strains. We have recently shown that *E. faecalis* uses the Ebp pilus, a heteropolymeric surface fiber, to bind the host protein fibrinogen as a critical step in CAUTI pathogenesis. Fibrinogen is deposited on catheters due to catheter-induced inflammation and is recognized by the N-terminal domain of EbpA (EbpA^NTD^), the Ebp pilus’s adhesin. In a murine model, vaccination with EbpA^NTD^ confers significant protection against CAUTI. Here, we explored the mechanism of protection using passive transfer of immune sera to show that antisera blocking EbpA^NTD^-fibrinogen interactions not only is prophylactic but also can act therapeutically to reduce bacterial titers of an existing infection. Analysis of 55 clinical CAUTI, bloodstream, and gastrointestinal isolates, including *E. faecalis*, *E. faecium*, and vancomycin-resistant enterococci (VRE), revealed a diversity of levels of EbpA expression and fibrinogen-binding efficiency *in vitro*. Strikingly, analysis of 10 strains representative of fibrinogen-binding diversity demonstrated that, irrespective of EbpA levels, EbpA^NTD^ antibodies were universally protective. The results indicate that, despite diversity in levels of fibrinogen binding, strategies that target the disruption of EbpA^NTD^-fibrinogen interactions have considerable promise for treatment of CAUTI.

## IMPORTANCE

Urinary catheterization is a routine medical procedure, and it has been estimated that 30 million Foley catheters are used annually in the United States. Importantly, placement of a urinary catheter renders the patient susceptible to developing a catheter-associated urinary tract infection, accounting for 1 million cases per year. Additionally, these infections can lead to serious complications, including bloodstream infection and death. *Enterococcus* strains are a common cause of these infections, and management of enterococcal infections has been more difficult in recent years due to the development of antibiotic resistance and the ability of strains to disseminate, resulting in a major threat in hospital settings. In this study, we developed an antibiotic-sparing treatment that is effective against diverse enterococcal isolates, including vancomycin-resistant enterococci, during catheter-associated urinary tract infections.

## INTRODUCTION

It is estimated that 20% to 50% of all hospitalized patients receive a urinary catheter (1, 2), placing them at risk for developing a catheter-associated urinary tract infection (CAUTI) (3). Short-term urinary catheterization increases the risk of developing CAUTI and other complications up to 80%, and prolonged catheterization can increase the risk to 100% (4–6). CAUTI is the most common cause of health-care-associated infection (HAI) worldwide, accounting for 40% of all HAIs (7, 8), and often leads to secondary bloodstream infection, with a 7-day mortality rate of more than 30% (7, 9–11). Current guidelines recommend antibiotic treatments lasting 7 to 14 days to prevent CAUTI (8, 12); however, control of CAUTIs has become a major challenge due to the development and dissemination of antibiotic resistances among the bacteria that cause HAI (9, 10).

A prominent example comes from bacteria in the genus *Enterococcus*, which have emerged as a leading cause of HAI and CAUTI (13, 14). Enterococcal HAI isolates are most commonly *Enterococcus faecalis* and *Enterococcus faecium*, whose ability to withstand heat, UV radiation, and aseptic solutions (14–16) allows them to persist in the hospital ecology (14, 16). Treatment is increasingly challenging because of their intrinsic and acquired resistance to recently introduced antibiotics (15–17), and vancomycin-resistant enterococci (VRE) along with multiply resistant enterococcus (MRE) strains are now common in CAUTI (18). Complete recovery from VRE infection may be prolonged and in some cases requires >3 years (19). Currently, 30% of all enterococcal HAI isolates are resistant to vancomycin (20), leading the Centers for Disease Control (CDC) to classify VRE as a serious threat, recommending continuous monitoring and the development of new therapeutic strategies (20).

New and improved treatment strategies will come from unraveling the host and bacterial factors that shift enterococci from commensalism to a pathogenic lifestyle. In this regard, we have recently found that urinary catheterization in both mice and humans elicits bladder inflammation, resulting in the release of fibrinogen into the bladder lumen (21) which then becomes deposited onto the catheter (22, 23). In a murine model of CAUTI, we found that released fibrinogen is critical for *E. faecalis* pathogenesis since (i) fibrinogen is used as a nutrient to promote enterococcal growth and (ii) *E. faecalis* exploits the fibrinogen-coated catheters to form biofilms. In the absence of fibrinogen, the bacterium cannot bind directly to the catheter material (23). *E. faecalis* expresses hair-like fibers called Ebp pili that are tipped with a fibrinogen-binding adhesin, EbpA, which binds directly to fibrinogen via its N-terminal domain (EbpA^NTD^). Immunization with EbpA^NTD^, but not immunization with whole pili, the EbpA C-terminal domain (EbpA^CTD^), or other pilus subunits, protects against CAUTI, reducing both catheter and bladder bacterial burdens (23). Furthermore, protection correlated with the production of antibodies that inhibit EbpA^NTD^-fibrinogen binding in several *in vitro* assays (23).

In this study, we evaluated the potential of EbpA^NTD^-based immunotherapies for translation to treatment of human CAUTI. The contribution of EbpA to CAUTI pathogenesis caused by a broad range of *E. faecalis* and *E. faecium* clinical isolates, the contribution of fibrinogen binding to biofilm formation on catheters recovered from human CAUTI, and the efficacy of EbpA^NTD^-based immunotherapy for treatment of CAUTI caused by a diverse collection of enterococcal clinical isolates were examined. Our results indicate that EbpA^NTD^-based immunotherapy is broadly effective and suggest that this approach would be effective for other enterococcal infections where fibrinogen is present.

## RESULTS

### *E. faecalis* colocalizes with fibrinogen during human CAUTI.

**FIG 1:**
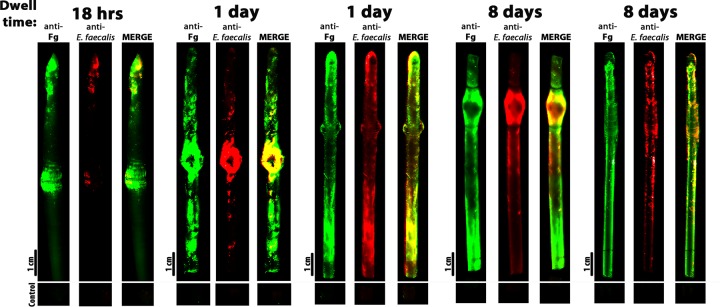
*E. faecalis* colocalized with Fg on human urinary catheters. Urinary catheters with an indwelling time of 18 h (A), 24 h (B and C), 8 days (D), or 9 days (E) were recovered from patients with an enterococcal UTI. The presence and distribution of bacteria and fibrinogen were assessed by immunofluorescence using antibody staining to detect fibrinogen (anti-Fg; green) and *E. faecalis* (anti-group D; red). As a negative control, a piece of the catheter was incubated with the secondary antibody only to assess background fluorescence.

To explore the role of the *E. faecalis*-fibrinogen interaction in human CAUTI, we examined the distribution of fibrinogen and *E. faecalis* on catheters recovered from human CAUTI. The catheters were obtained from patients undergoing both urological and nonurological procedures who developed an *E. faecalis*-positive urine culture prior to or after the placement of the catheter. Catheters were removed at different indwelling times and were immediately treated with fixative and then analyzed by fluorescence microscopy to assess patterns of fibrinogen deposition and enterococcal binding. This examination revealed both extensive but nonuniform distribution of fibrinogen (anti-Fg; Fig. 1) and extensive colonization of the catheter by *E. faecalis* (anti-*E. faecalis* [anti-group D]) (Fig. 1). Furthermore, *E. faecalis* localized only to regions with deposited fibrinogen (MERGE, Fig. 1), consistent with a role for fibrinogen in promoting *E. faecalis* adherence and biofilm formation on catheters.

### Passive transfer of EbpA^NTD^ antibodies prevents CAUTI.

**FIG 2:**
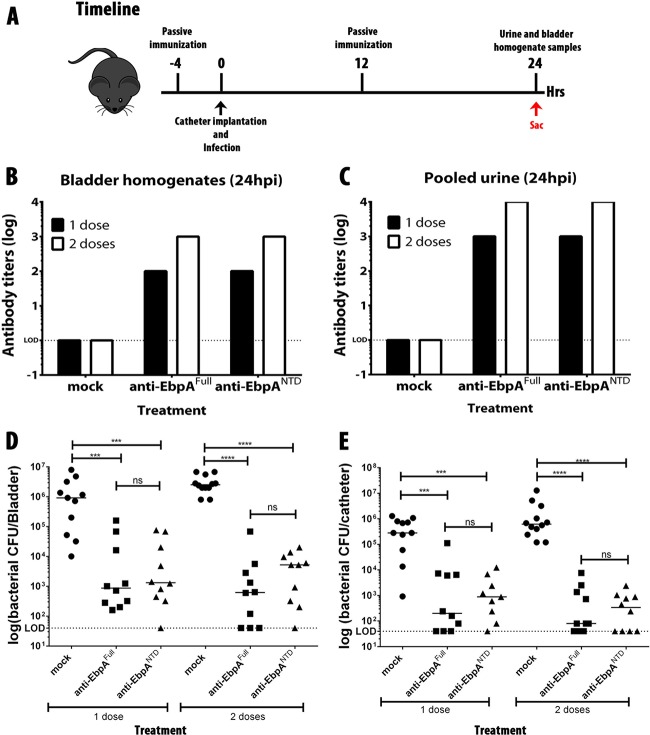
Passive immunization with anti-EbpA^Full^ and anti-EbpA^NTD^ antibodies prevents *E. faecalis* CAUTI. Mice (*n* = 10) were given a dose of 100 µl of PBS sera or 100 µl of anti-EbpA^Full^ or anti-EbpA^NTD^ with a titer of 1 × 10^7^. (A) Experimental timeline. Sac, time of mouse sacrifice. (B and C) Detection of EbpA^Full^ and EbpA^NTD^ antibodies in bladder (B) and in urine (C) analyzed by diluting each sample 1:100 before serial dilution. (D and E) Prophylaxis treatment performed using 1 dose at 4 h prior infection (D) or 2 doses at 4 h prior infection and 12 h postinfection (hpi) (E). Values represent means ± SEM. The Mann-Whitney *U* test was used for mouse experiments; *P* < 0.05 was considered statistically significant. *, *P* < 0.05; **, *P* < 0.005; ***, *P* < 0.0005; ****, *P* < 0.00005; ns, values were not statistically significantly different. The horizontal bar represents the median value. The horizontal broken line represents the limit of detection (LOD) of viable bacteria. Animals that lost the catheter were not included in this work.

Vaccination with EbpA or EbpA^NTD^ protects mice from CAUTI by *E. faecalis* OGIRF (23). Protection correlates with the development of serum antibody that can block EbpA-fibrinogen binding (23), suggesting an effector role for blocking antibody in protection. This hypothesis was tested by passive transfer of antisera to naive mice that were then implanted with catheters and challenged with *E. faecalis*. Mice received either 1 intraperitoneal (i.p.) dose of antisera at 4 h prior to catheter implantation and infection (ci) or doses at 12 and 4 h prior to ci (Fig. 2A). Antiserum was from mice immunized with EbpA or with EbpA^NTD^ or was serum from mice mock vaccinated with phosphate-buffered saline (PBS). Treated mice were then implanted with catheters and challenged with 2 × 10^7^ CFU of *E. faecalis* OG1RF. After 24 h postinfection (hpi), urine samples were collected, and mice were sacrificed to harvest bladders and catheters (Fig. 2A). Titration of bladder homogenates and urine indicated that anti-EbpA antibodies were present following treatment with sera from immunized mice but not but not following treatment with sera from mock-immunized mice and that their levels were higher in the mice that received 2 doses (Fig. 2B and C). Treatment with either anti-EbpA^Full^ or anti-EbpA^NTD^ antisera significantly reduced the mean bacterial burdens in the bladder (Fig. 2D) and catheter (Fig. 2E) of the treated mice by ~3 logs compared to mice receiving serum from mock-vaccinated mice (*P* < 0.005). Anti-EbpA^Full^ and anti-EbpA^NTD^ treatments were equally effective, and those mice that received a second dose of immune sera were more efficiently protected, with a reduction of mean bladder and catheter titers of ~4 logs compared to control mice (*P* < 0.0005). These data show that the serum antibody targeting EbpA^NTD^ is the immune effector conferring protection in EbpA-immunized mice.

### Catheterization facilitates release of antibodies into the bladder and urine.

**FIG 3:**
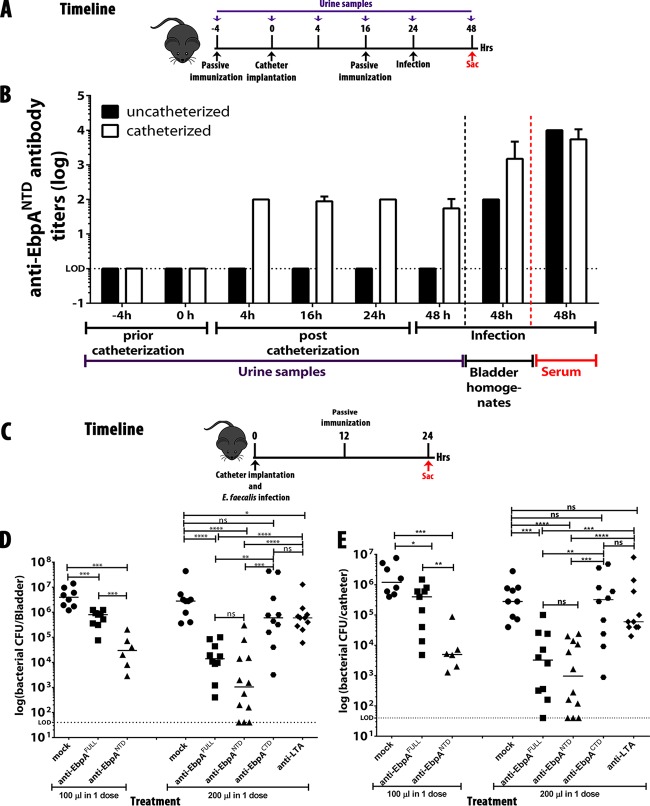
Release of anti-EbpA^NTD^ antibodies into the bladder and urine is mediated by bladder inflammation upon catheterization, and passive transfer of anti-EbpA^NTD^ antibodies reduced bacterial titers of an existing *E. faecalis* infection. (A) Experimental timeline. (B) Detection of anti-EbpA^NTD^ antibodies in urine samples, bladder homogenates, and serum samples was analyzed by diluting each sample 1:100 before serial dilution. (C) Experimental timeline. Mice (*n* = 10) were implanted with catheters and challenged with 1 × 10^7^ CFU of *E. faecalis* OG1RF. A dose of PBS serum, anti-EbpA^Full^, anti-EbpA^NTD^, anti-EbpA^CTD^, or anti-group D antibody was administered intraperitoneally (i.p.) at 12 h postinfection (hpi) (anti-EbpA^Full^, anti-EbpA^NTD^, anti-EbpA^CTD^, and anti-group D antibody titers of 1 × 10^7^). (D and E) Following 24 h of infection, bacterial burdens in bladder tissue (D) or recovered from catheters (E) were quantitated as the number of CFU recovered. Values represent means ± SEM. The Mann-Whitney *U* test was used; *P* < 0.05 was considered statistically significant. *, *P* < 0.05; **, *P* < 0.005; ***, *P* < 0.0005; ns, values were not statistically significantly different. The horizontal bar represents the median value. The horizontal broken line represents the limit of detection of viable bacteria. Animals that lost the catheter were not included in this work.

Serum antibody typically is not present at high concentrations in the bladder or urine. However, catheterization induces bladder inflammation, suggesting that, similarly to the release of fibrinogen (21, 23), catheter-induced inflammation also promotes the release of serum antibody into the bladder lumen. To assess this, we compared the levels of release of anti-EbpA^NTD^ antibodies into the bladders of uncatheterized and catheterized mice at several time points prior to and after catheter implantation. Titers were also determined following challenge with *E. faecalis* OG1RF (timeline, Fig. 3A). While anti-EbpA^NTD^ antibody was present in the serum of both catheterized and uncatheterized mice, antibody was detected in urine only following implantation of catheters (Fig. 3B) and was present at the earliest time point tested (4 h postcatheterization; Fig. 3B). Following infection, anti-EbpA^NTD^ antibodies were detected in bladder homogenates from both catheterized and uncatheterized mice; however, titers were lower by ~2 logs in the uncatheterized group (Fig. 3B). Taken together, these data show that catheterization and not infection is the major factor resulting in the release of serum antibody into the bladder.

### Anti-EbpA^NTD^ antibody reduces titers of a preexisting infection.

The therapeutic potential of anti-EbpA^NTD^ antibodies was explored by testing whether passive transfer of immune serum could reduce bacterial titers of a preexisting CAUTI. Mice were implanted with catheters and challenged with *E. faecalis* OG1RF, and infected mice were treated at 12 hpi with a single i.p. dose of 100 or 200 µl of a selected antiserum (timeline, Fig. 3C), including anti-EbpA^Full^, anti-EbpA^NTD^, anti-EbpA^CTD^ sera, serum from PBS-mock-vaccinated mice, or an antiserum (anti-*Streptococcus* group D antigen) that recognizes lipoteichoic acid (LTA), an abundant but unrelated surface antigen. At 24 h post-antibody treatment, mean bladder (Fig. 3D) and urine (Fig. 3E) CFUs were significantly reduced by treatment with anti-EbpA^Full^ or anti-EbpA^NTD^ sera but not by treatment with anti-EbpA^CTD^, mock sera, or anti-group D antigen antisera (*P* < 0.0005). Therapeutic efficacies were more pronounced in mice treated with the higher doses of EbpA^Full^ and anti-EbpA^NTD^ antisera (Fig. 3D and E). These results demonstrate that anti-EbpA antibodies can clear a preexisting infection, suggesting that the spectrum of anti-EbpA^NTD^-based immunotherapies can be expanded to include therapeutic options. In addition, the fact that antibodies targeting LTA were not protective emphasizes the unique vulnerability of EbpA^NTD^, validating EbpA-fibrinogen binding as a key step in enterococcal pathogenesis.

### *E. faecalis* infection does not confer protection against subsequent infection.

**FIG 4:**
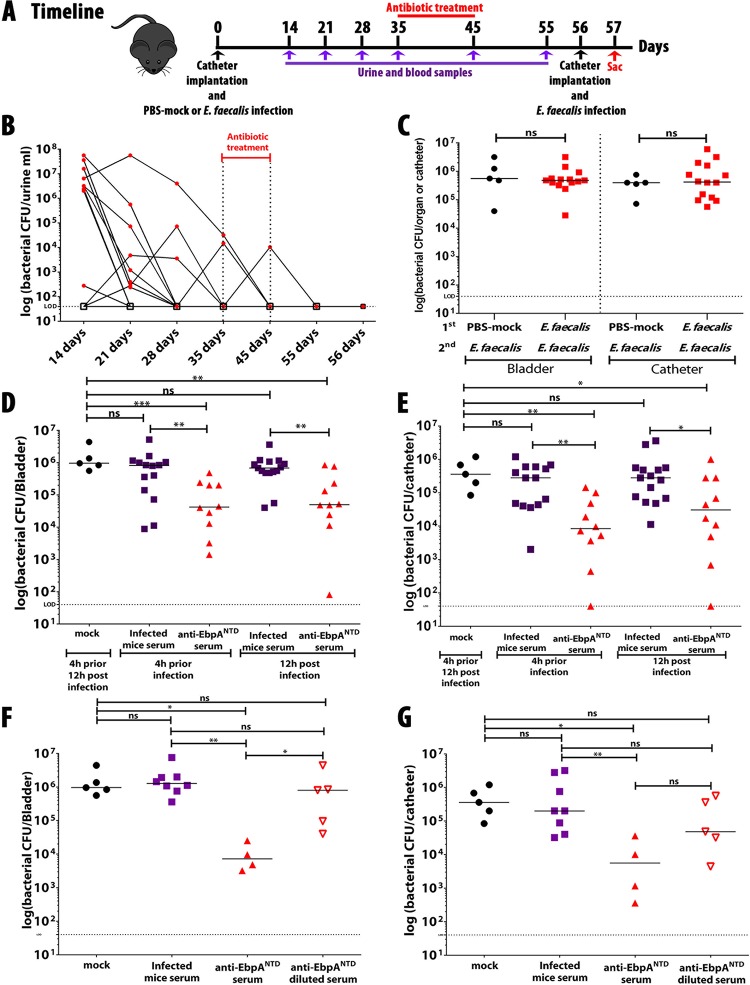
A prior enterococcal infection does not confer protection against a subsequent infection. (A and B) Mice were mock infected (*n* = 5) or infected with *E. faecalis* OG1RF (*n* = 15) (A); infection was followed by measuring the bacterial burden in urine (B). (C) After antibiotic treatment, mice were infected and bacterial burdens in bladder tissue or recovered catheters were quantitated as the number of CFU recovered. (D to G) Mice were immunized with mock serum (*n* = 5), serum from infected mice (*n* = 15 [D and E] or *n* = 10 [F and G]), anti-EbpA^NTD^ antibodies (*n* = 15 [D and E] or *n* = 5 [F and G]), or diluted anti-EbpA^NTD^ antibodies (*n* = 5). Following 24 h of infection, bladders (D and F) and catheters (E and G) were harvested and bacterial burdens were quantified. Values represent means ± SEM. The Mann-Whitney *U* test was used; *P* < 0.05 was considered statistically significant. *, *P* < 0.05; **, *P* < 0.005; ***, *P* < 0.0005; ns, values were not statistically significantly different. The horizontal bar represents the median value. The horizontal broken line represents the limit of detection of viable bacteria. Animals that lost the catheter were not included in this work.

Hospitalized patients with an enterococcal infection developed antibodies that recognized the Ebp pilus and its individual structural subunits (24, 25). However, it has not been demonstrated that these antibodies are protective against a subsequent infection. To assess this, mice were implanted with catheters and infected with *E. faecalis* OG1RF or were mock infected with PBS. To maximize the immune response and to prevent expulsion of the catheter and the resulting loss of infection, mice were not manipulated for the first 14 days hpi. Then, starting on day 14, urine and blood samples were collected every 7 days or as otherwise indicated (timeline, Fig. 4A) to assess levels of bacterial CFU (Fig. 4B) and production of anti-*E. faecalis* and anti-EbpA^NTD^ antibodies (see Fig. S1A and B in the supplemental material). At day 35, mice were treated with vancomycin (0.5 g/liter in drinking water) for 10 days and the clearance of CAUTI was monitored by assessment of urine CFU, which became undetectable by day 56 (Fig. 4B). At that time (Fig. 4A), mice were implanted with an additional catheter and were rechallenged with the original *E. faecalis* strain (OG1RF). When examined at 24 hpi, mean CFU levels in bladder and mean CFU levels on catheters were not significantly different between mice previously infected by *E. faecalis* and those that were mock infected (Fig. 4C), despite the fact that the former group developed antibodies against *E. faecalis* and EbpA^NTD^ (see Fig. S1A and B). However, the antibody titers were ~3 logs lower than those developed in EbpA^NTD^-vaccinated mice (see Fig. S1A and B) or in rabbits conventionally vaccinated with *E. faecalis* or EbpA^CTD^ (26). Furthermore, in contrast to serum from EbpA^NTD^-vaccinated mice, passive transfer of serum collected from infected mice neither prevented (Fig. 4D) nor treated (Fig. 4E) *E. faecalis* CAUTI and was no more effective than serum from mice mock vaccinated with PBS (Fig. 4D and E). Since it seemed likely that this failure to provide protection was correlated with the relatively low serum anti-EbpA^NTD^ titers that had developed during infection versus active vaccination, serum from EbpA^NTD^-vaccinated mice was diluted to a titer equivalent to that obtained by infection (1 × 10^−4^) (see Fig. S1C). The diluted serum from vaccinated mice was unable to protect against infection and failed to reduce bladder (Fig. 4F) and catheter (Fig. 4G) titers any better than serum from infected mice or from mice mock vaccinated with PBS. This result confirms that the anti-EbpA^NTD^ antibody concentration is crucial for protection and suggests that anti-EbpA^NTD^ antibodies generated in infected hospitalized patients may not be protective against a subsequent infection.

### The EbpA sequence is highly conserved across multiple enterococcal species.

**FIG 5:**
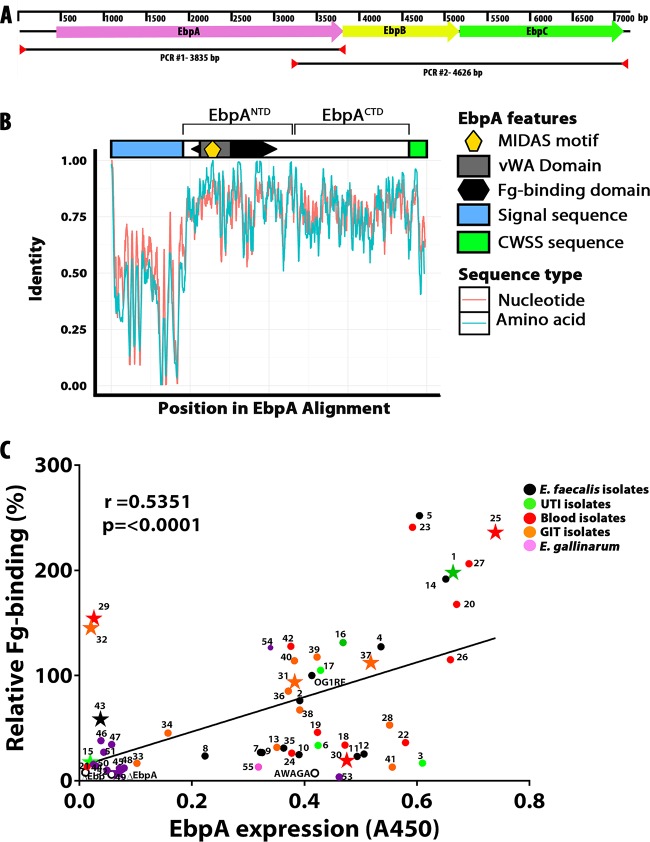
Ebp pilus operon presence and analysis of EbpA conservation and expression and of its function in binding to Fg in clinical enterococcal strains. (A) Ebp operon scheme. (B) Alignment of 137 unique EbpA peptide and nucleotide sequences by using a hidden Markov model. vWA, von Willebrand factor A. (C) Pearson correlation statistical analysis was performed to measure the correlation between EbpA expression and Fg binding of each tested enterococcal strain (*n* = 3). Each dot represents the average of results from two independent experiments, each consisting of 3 biological replicates. As a negative control for the expression of EbpA, ΔEbp pilus and ΔEbpA strains were used, and as a negative control for Fg binding, the EbpA MIDAS mutant strain (AWAGA) was used. The star symbols indicate the strains that were selected for additional *in vivo* analyses.

An ideal antivirulence therapeutic target should be conserved in carriage and sequence across many pathogenic strains and species. In this regard, the Ebp pilus has been reported to be widely distributed across the members of the genus *Enterococcus* (24, 27). To extend these observations, we examined a diverse collection of 55 isolates to assess the presence and conservation of the Ebp pilus operon in clinical strains (see Table S1 in the supplemental material). This analysis revealed that all three genes that comprise the Ebp pilus were present in all 55 enterococcal genomes analyzed (Fig. 5A; see also Fig. S2A). Expanding this analysis to a collection of genome sequences available in public databases representing multiple enterococcal species, a hidden Markov model was used to search for EbpA homologous sequences. A total of 480 peptides matching EbpA were identified from eight *Enterococcus* species, including *E. faecalis*, *E. faecium*, *E. gallinarum*, *E. saccharolyticus*, *E. mundtii*, *E. hirae*, *E. casseliflavus*, and *E. flavescens*. After filtering for unique sequences and removal of sequences with low coverage (<80%) of the EbpA open reading frame were performed, the resulting 137 unique peptide and nucleotide sequences were aligned (Fig. 5B). The resulting gapped alignment shows that the sequences representing the first ~100 amino acids of EbpA, comprising mainly the signal sequence, are highly divergent between species of *Enterococcus*, although they are generally well conserved within species (e.g., *E. faecalis* peptide sequences share 99.6% average pairwise identity in the first 100 amino acids compared to 46.8% average pairwise identity in the first 100 amino acids in all *Enterococcus* EbpA peptide sequences). In contrast, the sequences of the EbpA N-terminal domain (NTD) and C-terminal domain (CTD) are well conserved across the members of the *Enterococcus* genus, with an average pairwise identity of 73.7%, including 100% peptide sequence identity of the EbpA^NTD^ MIDAS motif (Fig. 5B) that is required for binding to fibrinogen (23). This high degree of sequence similarity predicts conservation of the protective epitopes recognized by anti-EbpA^NTD^ antibodies raised against EbpA of *E. faecalis* OG1RF.

### Surface-expressed EbpA correlates with fibrinogen binding among enterococcal strains.

Conservation of EbpA epitopes predicts that an EbpA antisera raised against one strain will recognize EbpA expressed by diverse strains. This prediction was tested using antisera raised against EbpA of *E. faecalis* OG1RF and a panel of clinical strains isolated from the urinary tract (UT),the bloodstream, and the gastrointestinal tract (GIT), including representatives of *E. faecalis*, *E. faecium*, *E. gallinarum*, and VRE, as well as several unclassified enterococcal isolates (see Table S1 in the supplemental material). Expression of cell surface EbpA was evaluated by enzyme-linked immunosorbent assay (ELISA) following *in vitro* culture under conditions known to promote Ebp pilus expression in OG1RF (static culture in brain heart infusion [BHI] medium for 18 h at 37°C). It was found that the EbpA antiserum detected surface-expressed EbpA in all strains tested (see Fig. S2A); however, there was a considerable range in the amount of EbpA that was detected across the panel of isolates (see Fig. S2B). To evaluate whether this range reflected differences in the efficiency by which the antiserum recognized EbpA from different strains versus differences in the levels of Ebp pilus expression, the panel of isolates was tested for fibrinogen binding. This analysis also revealed a range of binding efficiencies of the various isolates (see Fig. S2C). However, there was a positive (*r* = 0.5385) and significant (*P* < 0.0001) correlation between the detected levels of surface-expressed EbpA and the levels of fibrinogen bound (Fig. 5C). Correlation between detection and function suggests that there is heterogeneity in Ebp expression between isolates rather than differential abilities of the anti-EbpA antiserum to detect heterologous EbpA proteins. Taken together, these data show that EbpA is a ubiquitous factor expressed among enterococcal strains and species and that it has a conserved antigenic profile.

### EbpA^NTD^-based immunotherapies can prevent and treat CAUTI caused by diverse enterococcal isolates.

**FIG 6:**
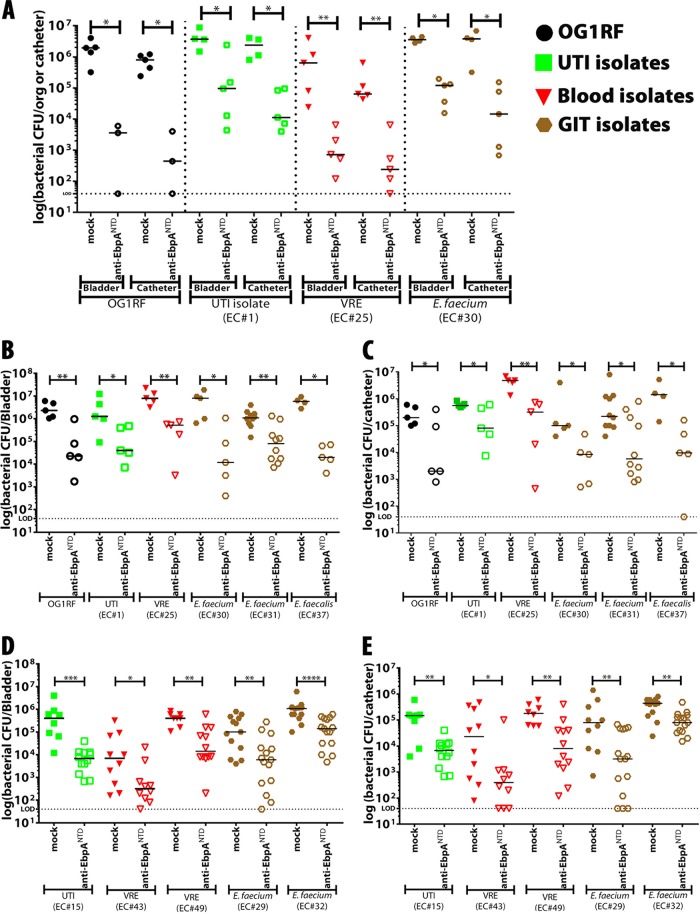
EbpA^NTD^-based vaccine and anti-EbpA^NTD^ antibody treatment prevented and reduced bacterial titers of different enterococcal isolates. (A) Mice were immunized and received two booster immunizations with doses of 100 µg EbpA^NTD^. Four weeks following the final immunization, mice were implanted with catheters and challenged with 2 × 10^7^ CFU of *E. faecalis* OG1RF (*n* = 5) or with the indicated clinical enterococcal strains (*n* = 5). Following 24 h of infection, bacterial burdens in bladder tissue or recovered catheters were quantitated as the number of CFU recovered. org, organ. (B to E) To assess the therapeutic effect of anti-EbpA^NTD^ antibodies against enterococcus-infected mice, antibodies were administered intraperitoneally 12 hpi (anti-EbpA^NTD^ antibody titer of 1 × 10^7^). Enterococcal strains were divided into two groups: (i) those that highly expressed EbpA and bound to Fg (B and C; *n* = 5) and (ii) those that did not express EbpA (*n* = 10 [EC#15 and EC#43] and *n* = 15 [EC#49]) or bind to Fg (D and E; *n* = 15) under *in vitro* conditions. Bladder (B) and catheter (C) bacterial burdens were quantified as described below. Values represent means ± SEM. The Mann-Whitney *U* test was used; *P* < 0.05 was considered statistically significant. *, *P* < 0.05; **, *P* < 0.005; ***, *P* < 0.0005; ns, values were not statistically significantly different. The horizontal bar represents the median value. The horizontal broken line represents the limit of detection of viable bacteria. Animals that lost the catheter were not included in this work.

The conservation of EbpA epitopes suggests that EbpA^NTD^-based immunotherapies would be effective against CAUTI caused by a wide range of enterococcal strains and species. To test this, mice were immunized with the OG1RF EbpA^NTD^-based vaccine using a standard protocol (100 µg purified EbpA^NTD^ emulsified in Freund’s complete adjuvant, with booster immunizations corresponding to the original dose on weeks 4 and 8) or were subjected to mock vaccination with PBS in adjuvant, as described previously (23). Mice were challenged by a panel of strains selected to have high levels of surface-expressed EbpA and of fibrinogen binding that also reflected clinical diversity, including a UTI isolate (EC#1), a VRE blood isolate (EC#25), and an *E. faecium* GIT isolate (EC#30) (see Table S1 in the supplemental material) (Fig. 5A; see also Fig. S2A and B). Efficacy of protection was benchmarked against infection by *E. faecalis* OG1RF. Four weeks after the second boost, mice were implanted with catheters and challenged with 2 × 10^7^ CFU of the indicated strains. Analyzed at 24 hpi, it was found that all tested strains were able to colonize the bladder and the catheter (Fig. 6A). Furthermore, EbpA^NTD^ vaccination significantly reduced bladder and catheter burdens versus the levels seen with mock-vaccinated mice for all strains examined (Fig. 6A).

Examined next was whether passive transfer of anti-EbpA^NTD^ antibodies could treat CAUTI by reducing bacterial titers of an ongoing infection. For this analysis, the number of clinical strains tested was expanded to include those showing (i) high EbpA expression and high fibrinogen binding levels (EC#1, EC#25, EC#30, EC#31, and EC#37); (ii) low EbpA expression and low fibrinogen binding levels (EC#15, EC#43, and EC#49); and (iii) a low EbpA expression level but a high fibrinogen binding level (EC#29 and EC#32) (Fig. 5C; see also Fig. S2A and B in the supplemental material). As before, these strains represented UTI, blood, and GIT isolates. Mice were catheterized and challenged with 2 × 10^7^ CFU of the indicated strains; then, mice received 1 dose of 200 µl of anti-EbpA^NTD^ or PBS-(mock)-vaccinated serum at 12 hpi. Examined at 24 hpi, all strains that highly expressed EbpA were found to have colonized the bladder and catheter with CFU levels equivalent to or higher than those measured for *E. faecalis* OG1RF (Fig. 6B and C) and that treatment with EbpA^NTD^ antibodies reduced bacterial titers by ~3 to 4 logs compared to the levels seen with sera from control mice subjected to mock treatment with PBS (Fig. 6B and C). Those strains that expressed low levels of EbpA *in vitro* were also able to cause CAUTI and to colonize the bladder and catheter (Fig. 6D and E); however, their colonization was not as efficient (10^4^ to 10^5^ CFU/ml) as that seen with OG1RF and the strains expressing high levels of EbpA (10^6^ to 10^7^ CFU/ml). Analysis of two strains (EC#29 and EC#32) that showed anomalous binding behavior *in vitro* (high levels of binding of Fg with only low-level expression of EbpA) revealed that they were also able to colonize the bladder and catheter (10^5^ to 10^6^ CFU/ml). However, despite their low levels of EbpA expression *in vitro*, treatment with anti-EbpA^NTD^ antibodies was an effective therapy, reducing catheter and bladder CFUs ~1 to ~1.5 logs compared to PBS mock treatment serum controls (Fig. 6B and C). Overall, these data show that EbpA^NTD^-based immunotherapies have efficacy against a broad range of isolates.

## DISCUSSION

In this study, we showed that passive immunization with anti-EbpA^NTD^ antibodies is effective both as a prophylactic and as a therapy for the reduction of bacterial titers in a murine CAUTI model and that the level of protection correlated with the concentration of anti-EbpA^NTD^ antibodies. More importantly, this therapy was also protective against a broad range of urinary tract, bloodstream, and GIT enterococcal clinical strains, which included *E. faecalis*, *E. faecium*, and VRE. Our observation that, during human CAUTI, *E. faecalis* colocalized with Fg deposited onto a urinary catheter recapitulated results from the murine CAUTI model and from *ex vivo* analyses with catheters removed from infected humans (28, 29) and suggests that EbpA^NTD^-based immunotherapies can be directly translatable to treatment of human disease.

Since it has been shown that EbpA is required for infection in the murine model (23) and that mutation of the EbpA MIDAS motif blocks both Fg binding and virulence (23, 30), it is likely that EbpA-Fg interactions represent a key step in CAUTI pathogenesis by directing initial adherence to the catheter surface. This is supported by studies showing colocalization of catheter Fg and bound enterococci (22, 23). Thus, the most parsimonious model for protection would be via a steric mechanism where antibodies that block binding to Fg prevent this initial attachment event. However, since passive immunization could act therapeutically against an established infection, the mechanism of protection may be more complex. Attachment and biofilm formation may be dynamic, involving multiple cycles of attachment and detachment. Thus, when detached, enterococci may become susceptible to antibodies that block reattachment. Alternatively, a multifactorial mechanism could involve an EbpA-dependent opsonic activity or an unknown EbpA activity in a domain adjacent to its Fg binding site. Therefore, it will be of great interest to characterize the basis of EbpA^NTD^-mediated protection.

On the basis of our bioinformatic analysis, we found that EbpA^NTD^ was highly conserved among diverse enterococcal species, suggesting that multiple enterococcal lineages use a common mechanism of CAUTI pathogenesis. This would explain the effectiveness of the EbpA^NTD^ immunotherapies observed in the present study. Other enterococcal species distributed across different clades of the *Enterococcus* genus phylogeny (31, 32) have been found to cause human infections, suggesting that these diverse species may also have common traits for colonization, and virulence, which may include EbpA.

Supporting the hypothesis of common mechanisms was the overall correlation between EbpA expression *in vitro* and Fg binding across a panel of 55 strains. However, there were exceptions, including two strains with a low level of EbpA expression but a high level of fibrinogen binding. That these atypical strains colonized bladder and catheter in the CAUTI model but were susceptible to EbpA^NTD^-based immunotherapy suggests that Ebp pili play a critical role in CAUTI pathogenesis that cannot be compensated for by other Fg-binding activities that can be expressed by enterococci, including Fss proteins and PrpA (25, 33). It is possible that heterogeneity in regulation of Ebp means that pilus expression *in vitro* may not reflect levels *in vivo*. There are many examples where *in vitro* conditions do not recapitulate *in vivo* conditions (34, 35). Thus, it will be important to understand the environmental cues in a catheterized bladder that play a role in Ebp pilus expression.

Antibiotic treatment is the standard of care for an enterococcal CAUTI but is increasingly unsuccessful. In fact, catheters often must be removed because the patient does not respond to even very rigorous antibiotic treatment. Treatment failure can have other collateral effects, including native flora dysbiosis, in which the elimination of gut commensals leads to the absence of microbe-derived products such as flagella that stimulate secretion of RegIIIγ and other host defense molecules. This allows enterococci to overgrow in the gut, causing inflammation, anastomotic leakage, and bloodstream infection (28, 36, 37). Furthermore, patients hospitalized for long periods under conditions of antibiotic treatment are susceptible to infection by *E. faecium*, which is frequently resistant to vancomycin and ampicillin (14, 16, 17) and under these conditions can become the dominant flora (16, 29). Thus, alternative therapies are needed to treat and to prevent the evolution of multiply resistant enterococci. Antibiotic-sparing therapies targeting enterococcus-Fg interaction may hold considerable promise, and since EbpA is highly conserved and important for colonization, it may represent the ideal target for this approach. Its critical role also suggests that enterococci must protect it from the immune response in order to cause persistent infections. Consistent with this, mice with *E. faecalis* CAUTI generated low titers of anti-EbpA^NTD^ antibodies, which did not confer resistance against reinfection. Since EbpA is a minor component of a large heteropolymer, it is unlikely to be the immunodominant component of the pilus fiber. A similar strategy of compartmentalizing the adhesive activity to a single subunit of a large heteropolymer is used by uropathogenic *Escherichia coli* to protect the adhesive subunit of its type 1 pilus adhesin (38). Alternatively, *E. faecalis* may further exploit its ability to interact with Fg to coat both individual cells and biofilm with a host protein in order to obtain immune privilege and evade recognition by the immune response. Thus, any strategy targeting EbpA must defeat the mechanisms that enterococci have evolved to protect this Achilles heel from the host’s protective responses. Fibrinogen is found in the bloodstream and is a marker of vascular rupture and responsible for coagulation, fibrosis, protection from infections, and other functions (39). Based on the importance of fibrinogen for *E. faecalis* colonization via EbpA and the conservation of EbpA among enterococcal strains and given that fibrinogen is present during in other situations as infective endocarditis, bacteremia, and intra-abdominal and surgical-site infections (40), it is feasible that fibrinogen could be a general factor for the pathogenesis of *Enterococcus* spp. It may also represent a common strategy for biofilm formation and colonization of catheters, indwelling devices, or tissues at other anatomical sites, including venous catheters, orthopedic implants, and damaged heart valves in endocarditis (41). Thus, EbpA-Fg interactions represent a vulnerability that can be exploited for intervention in enterococcal infections where Fg is present.

## MATERIALS AND METHODS

### Bacterial strains and growth conditions.

Unless otherwise specified, *E. faecalis* strain OG1RF and its derivatives and enterococcal strains were grown overnight on brain heart infusion broth (BHI) (BD Company) and were inoculated from a single bacterial colony grown on BHI agar plates. Liquid cultures were grown statically at 37°C for 18 h or as otherwise indicated. Bacterial strains are listed in Table S1 in the supplemental material.

### General cloning techniques.

Bacterial genomic DNA (gDNA) was isolated by using a Wizard genome DNA purification kit (Promega Corp). Primers were purchased from Integrated DNA Technology. Phusion High Fidelity DNA polymerase was purchased from New England Biolabs and used according to the methods described by the manufacturer.

### Antibodies used in this study.

The primary antibodies used in the study were rabbit anti-*Streptococcus* group D antigen (anti-*E. faecalis* lipoteichoic acid) (Lee Laboratories) (26) and mouse anti-EbpA^Full^, mouse anti-EbpA^NTD^, and mouse anti-EbpA^CTD^ (generated in this study). The secondary antibodies used in the study were IRDye 800CW donkey anti-goat (catalog no. 926-32213) and IRDye 680LT goat anti-rabbit (catalog no. 926-68021) antibodies from Li-Cor Biosciences and horseradish peroxidase (HRP)-conjugated goat anti-mouse and goat anti-rabbit antisera from KPL.

### Human urinary catheter analysis.

The human urinary catheter was collected per the study approval from the Washington University School of Medicine Internal Review Board (approval #201410058). After collection, the catheter was cut into 10-cm-long segments and the segments were fixed with formalin for 1 h and washed (3 times) with PBS. For analysis, the first 10 cm of the catheter’s tip was blocked by overnight incubation at 4°C with 1.5% bovine serum albumin (BSA)–0.1% sodium azide–PBS (BB) and then washed three times for 5 min each time with PBS-T (PBS containing 0.05% Tween 20). A 15-ml solution containing goat anti-human fibrinogen and rabbit anti-*Streptococcus* group D (1:500) antisera in dilution buffer (PBS with 0.05% Tween 20, 0.1% BSA, and 0.5% methyl α-d-mannopyronoside) was added, and the reaction mixture was incubated at room temperature for 2 h. Then, it was washed (3 times) for 5 min each time with PBS-T and incubated with donkey anti-goat IRDye 800CW (diluted 1:10,000) in dilution buffer for 45 min at room temperature. Following an additional 3 washes with PBS-T, catheters were examined for infrared signal using an Odyssey Imaging System (Li-Cor Biosciences). Autofluorescence was assessed in nonimplanted catheters and catheters not incubated with the primary antibody and was minimal.

### Mouse catheter implantation and infection.

The mice used in this study were 6-week-old female wild-type C57BL/6Ncr mice purchased from Charles River Laboratories. Mice were subjected to transurethral implantation and inoculated as previously described (34). Mice were anesthetized by inhalation of isoflurane and implanted with a 5-mm length of platinum-cured silicone catheter. When indicated, mice were infected immediately following catheter implantation with 50 µl of ~2 × 10^7^ CFU of bacteria in PBS introduced into the bladder lumen by transurethral inoculation as previously described (34). To harvest the catheters and organs, mice were sacrificed at the desired time points by cervical dislocation after anesthesia inhalation, and the bladders were aseptically harvested. Subsequently, the silicone implant was retrieved from the bladder, when present. All studies and procedures were approved by the Animal Studies Committee at the Washington University School of Medicine.

### Active and passive immunization of mice.

Mice were immunized as previously described (23). Groups of 10 mice were used for each vaccine dose. One hundred micrograms of EbpA proteins or PBS was emulsified with either Freund’s complete adjuvant (first vaccination) or Freund’s incomplete adjuvant (boosts). Mice were vaccinated intramuscularly and then with the same dose at 4 and 8 weeks. Mouse sera were collected prior to immunization and every week after to determine the antibody response. At 4 weeks after the second boost, mice were implanted with catheters and challenged with ~2 × 10^7^ CFU of *E. faecalis* OG1RF or clinical enterococcal isolates. Mice were sacrificed at 24 hpi to determine bacterial titers in the bladder and catheters, as described above. For the passive immunization experiments, the specified antiserum was administered via intraperitoneally injection. Specific antisera, concentrations, and doses are specified in the timeline for each experiment performed in this study. Control groups were administered PBS-mock vaccination serum or an antiserum targeting LTA (anti-*Streptococcus* group D antigen antiserum). Mice were subjected to catheter implantation and challenged as described before.

### Determination of antibody responses in mice.

A 100-µl volume of *E. faecalis* OG1RF at an optical density at 600 (OD_600_) of 0.5 or 10 µg of EbpA proteins was used to coat Immulon 4HBX flat-bottom microplates (Thermo, Fisher) overnight at 4°C. Plates were washed (3 times) with PBS-T to remove unbound bacteria or protein and blocked for 2 h with BB. Bladder homogenates and urine and serum samples from infected or vaccinated mice were diluted 1:100 in dilution buffer before serial dilutions. Then, 100 µl of the diluted samples was added into the plate and incubated for 2 h at room temperature. After the incubation, the plates were washed (3 times) with PBS-T, followed by a 1-h incubation with HRP-conjugated goat anti-mouse antisera (1:2,000), and were then washed (3 times) with PBS-T. Detection was performed using a TMB substrate reagent set (BD catalog no. 555214). The reaction mixtures were incubated for 5 min to allow color to develop, and the reactions were then stopped by the addition of 1.0 M sulfuric acid. The absorbance was determined at 450 nm. Titers were defined by the last dilution with an *A*_450_ of at least 0.2.

### Presence of Ebp pilus operon in enterococcal strains.

Bacterial strains were grown overnight in BHI broth, and gDNA was isolated by following the manufacturer’s instructions. The *ebp* pilus operon was PCR amplified by using two sets of primers. The sets of primers were designed from a highly conserved region that came from an alignment of 500 *ebp* operon nucleotide sequences from different *Enterococcus* species, including *E. faecalis*, *E. faecium*, *E. gallinarum*, *E. saccharolyticus*, *E. mundtii*, *E. hirae*, *E. casseliflavus*, and *E. flavescens*. The first set of primers, ALFM65-ALFM80, amplifies 3,835 bp from 422 bp upstream of the *ebpA* gene to the first 98 bp of *ebpB* (ALFM65 [F], 5′-GCAAGTTCTTTTTTAGTCATCCA-3′; ALFM80 [R], 5′-TGAACGCTTGCTTGCGATGCCTCTG-3′). The second set of primers, ALFM70-ALFM85, amplifies 4,626 bp from the last 552 bp of *ebpA* to 104 bp downstream of *ebpC* gene (ALFM70 [F], 5′-GGAAATTATGAATTTACTGTTGATAAATATGG-3′; ALFM85 [R], 5′-ACTTCATTGCTTCCTCC-3′). Each PCR product was visualized by using electrophoresis and 1% Tris-borate-EDTA (TBE) agarose gels.

### Expression of EbpA in the cell surface of enterococcal strains.

Surface expression of EbpA by clinical and laboratory enterococcal strains was detected by whole-cell ELISA as previously described (23). Bacterial strains were grown for 12 h in BHI broth, washed (3 times) with PBS, and normalized to an OD_600_ of 0.5. Then, bacterial cells were washed and resuspended with 50 mM carbonate buffer (pH 9.6) containing 0.1% sodium azide and 100 µl of the each bacterial solution was used to coat Immulon 4HBX microtiter plates overnight at 4°C. Plates were washed with PBS-T to remove unbound bacteria and blocked for 2 h with BB followed by washes performed with PBS-T. EbpA surface expression was detected using mouse anti-EbpA^Full^ antisera, which was diluted 1:100 in dilution buffer before serial dilutions were performed as described above. A 100-μl volume was added to the plate, and the reaction mixture was incubated for 2 h. After the incubation, the plates were washed with PBS-T followed by 1 h of incubation with HRP-conjugated goat anti-rabbit antisera (1:2,000) and were then washed with PBS-T. Detection was performed using a TMB substrate reagent set. The reaction mixtures were incubated, developed, and measured as described above. As an additional control, rabbit anti-*Streptococcus* group D antiserum was used to verify that whole cells of all strains were bound to the microtiter plates at similar levels.

### Whole-bacterial binding to fibrinogen.

To determine whether enterococcal strains adhere to immobilized fibrinogen as previously described (23), Immulon 4HBX microplates were coated overnight at 4°C with 100 µg/ml of human fibrinogen that was free of plasminogen and von Willebrand factor (Enzyme Research Laboratory). The plates were blocked for an hour with BB, followed with PBS washes (performed 3 times for 5 min each time). Bacterial strains were grown for 12 h in BHI broth, were normalized to an OD_600_ of 0.5, and then were washed and resuspended in PBS. A total of 100 µl of bacteria was added to the coated wells and incubated for an hour at 37°C, followed by PBS washes performed using the wash function of a microplate reader (ELX405 select CW; Biotek Instruments) to remove the unbound bacteria. Next, bacterial cells were fixed with formalin for 20 min at room temperature, followed by washes performed with PBS-T, and were then blocked overnight at 4°C with BB, and PBS-T washes were performed. Then, plates were incubated for an hour at room temperature with rabbit anti-*Streptococcus* group D antigen antisera (1:500). Plates were washed with PBS-T, incubated with Odyssey secondary antibody (goat anti-rabbit IRDye 680LT, diluted 1:10,000 in dilution buffer) for 45 min at room temperature, and washed with PBS-T. As a final step, the plates were scanned for infrared signal using an Odyssey imaging system (Li-Cor Biosciences).

### EbpA sequence analysis.

Using the amino acid sequence of *E. faecalis* OG1RF as a query, we identified homologs of EbpA in the UniProtKB database using the PHMMER webserver (42, 43). Nucleotide sequences of the genes encoding matches were then retrieved from the Ensembl database (44) using their unique identifiers. Homologous gene sequences were then filtered to remove duplicate nucleotide sequences and nucleotide sequences which did not cover >80% of the OG1RF gene sequence. Amino acid sequences of the unique EbpA homologs were then aligned using the MAFFT algorithm (45) and the BLOSUM62 scoring matrix, and a corresponding codon-based alignment of the nucleotide sequences was constructed using PAL2NAL (46). The percent identity of these alignments was then visualized using sliding windows of 20 amino acids and 60 nucleotides for the protein and gene sequences, respectively.

### Statistical analyses.

Data from multiple experiments were pooled. Two-tailed Mann-Whitney *U* tests were performed with GraphPad Prism 5 software (GraphPad Software, San Diego, CA) for all comparisons described for CAUTI experiments. Antibody titration, expression of EbpA, and binding assays were analyzed by using a paired *t* test to evaluate the significance of differences. To measure the strength of the linear relationship between EbpA expression and fibrinogen binding, a Pearson correlation analysis was performed. Values represent means ± standard errors of the means (SEM) derived from results of at least 3 independent experiments (*, *P* < 0.05; **, *P* < 0.005; ***, *P* < 0.0005; ****, *P* < 0.00005; ns, difference not statistically significant).

## SUPPLEMENTAL MATERIAL

Figure S1  Titration of anti-*E. faecalis* and anti-EbpA^NTD^ antibodies. (A and B) Titration of anti-*E. faecalis* (A) and anti-EbpA^NTD^ (B) antibodies from *E. faecalis*-infected mouse sera. (C) Dilution of anti-EbpA^NTD^ antibodies to levels comparable to those of EbpA^NTD^ titers from *E. faecalis* infected mice. Titers were analyzed by pooling samples from 10 individual mice in each immunization treatment and diluting the pooled samples 1:100 before serial dilution. Anti-*E. faecalis* and anti-EbpA^NTD^ antibodies were used as positive controls for the ELISA and mouse naive sera as negative controls. Download Figure S1, TIF file, 16.7 MB

Figure S2  (A) Clinical enterococcal strains were assessed for the presence of Ebp pilus by PCRs. (B) Expression of EbpA at the surface of the cells was assessed by coating ELISA plates with the strains. Mouse anti-EbpA^NTD^ was used to detect surface-expressed EbpA. (C) Adherence of the indicated whole bacterial strains to fibrinogen (Fg)-coated surfaces was assessed by ELISA using a rabbit anti-group D streptococcal antibody for detection of enterococcal bacteria. Download Figure S2, TIF file, 27.7 MB

Table S1  Laboratory and clinical enterococcal strains used. Table S1, DOCX file, 0.04 MB
