# Supplementary figures and images for: Antibody-Based Therapy for Enterococcal Catheter-Associated Urinary Tract Infections
Source: mBio. 2016 Oct 25;7(5):e01653-16. doi: 10.1128/mBio.01653-16 (PMC5080383; doi:10.1128/mBio.01653-16)

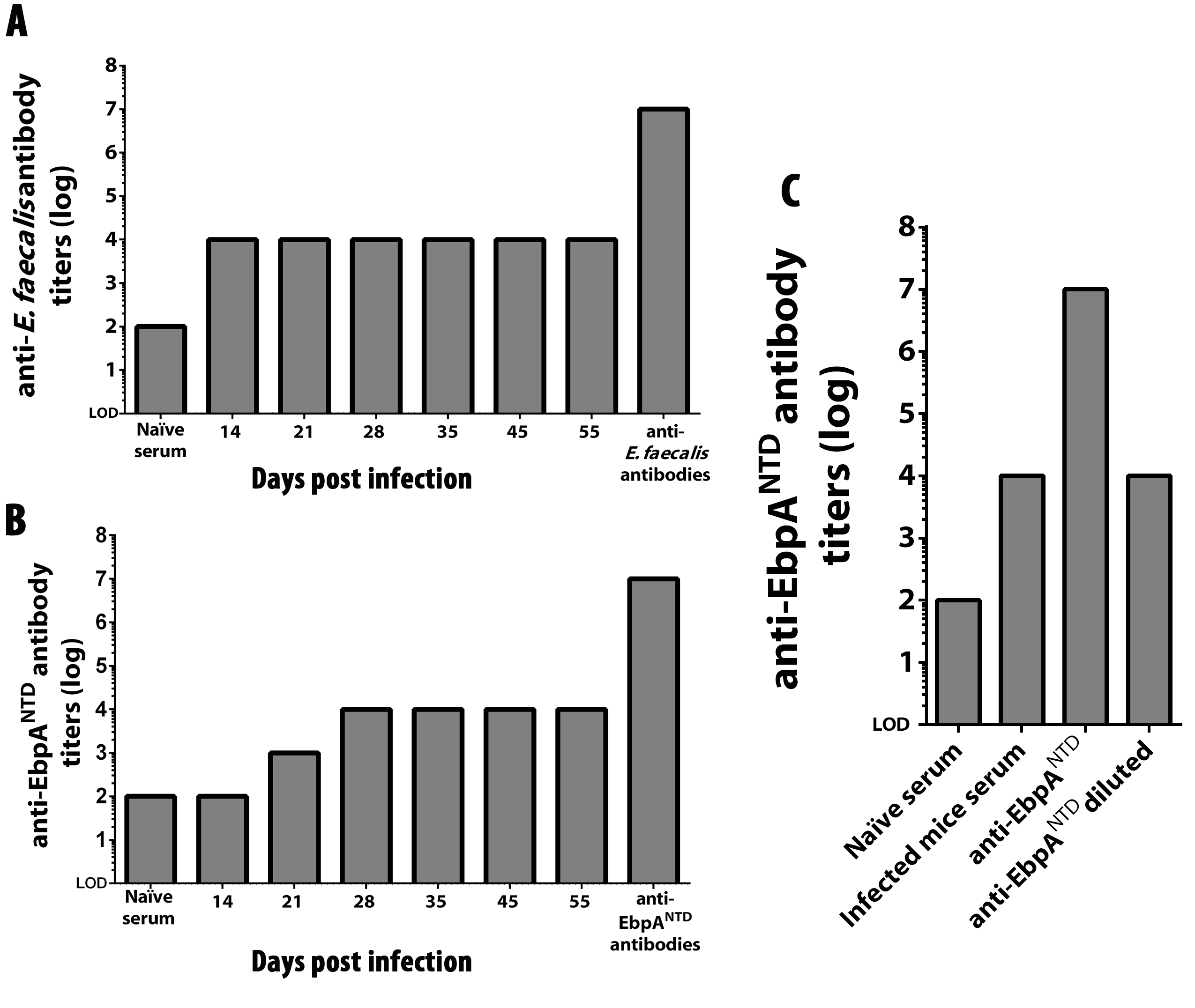

Supplement: Figure S1 — Titration of anti-E. faecalis and anti-EbpANTD antibodies. (A and B) Titration of anti-E. faecalis (A) and anti-EbpANTD (B) antibodies from E. faecalis-infected mouse sera. (C) Dilution of anti-EbpANTD antibodies to levels comparable to those of EbpANTD titers from E. faecalis infected mice. Titers were analyzed by pooling samples from 10 individual mice in each immunization treatment and diluting the pooled samples 1:100 before serial dilution. Anti-E. faecalis and anti-EbpANTD antibodies were used as positive controls for the ELISA and mouse naive sera as negative controls. Download [file mbo005163049sf1.tif]

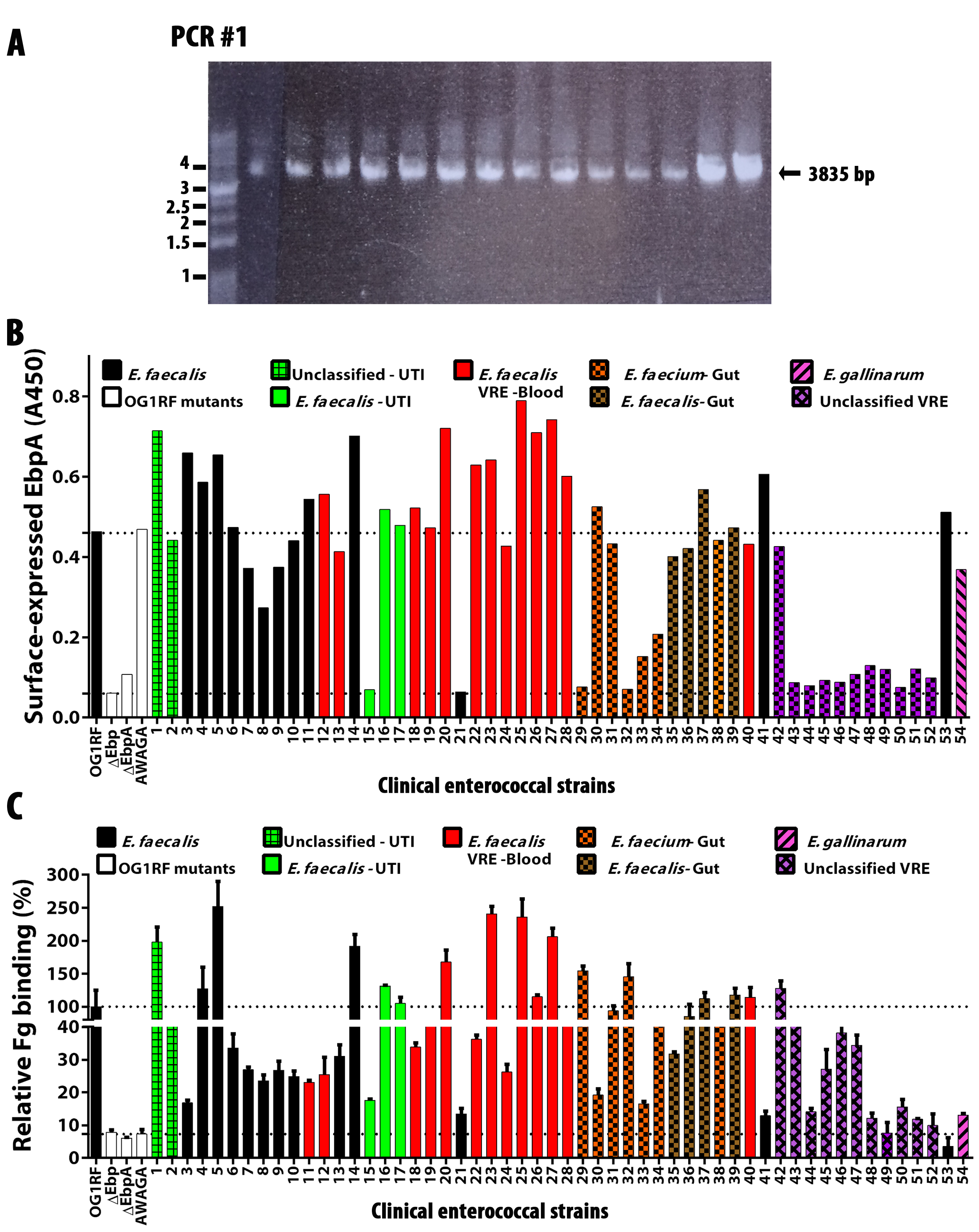

Supplement: Figure S2 — (A) Clinical enterococcal strains were assessed for the presence of Ebp pilus by PCRs. (B) Expression of EbpA at the surface of the cells was assessed by coating ELISA plates with the strains. Mouse anti-EbpANTD was used to detect surface-expressed EbpA. (C) Adherence of the indicated whole bacterial strains to fibrinogen (Fg)-coated surfaces was assessed by ELISA using a rabbit anti-group D streptococcal antibody for detection of enterococcal bacteria. Download [file mbo005163049sf2.tif]
