# Supplementary material for: Antibody-Based Therapy for Enterococcal Catheter-Associated Urinary Tract Infections
Source: mBio. 2016 Oct 25;7(5):e01653-16. doi: 10.1128/mBio.01653-16 (PMC5080383; doi:10.1128/mBio.01653-16)
Supplement: Table S1 — Laboratory and clinical enterococcal strains used. [file mbo005163049st1.docx]

**Supplementary Table 1. Laboratory and clinical enterococcal strains used**

| **Code** | **Catalog #** | **Specie** | **Description** | **Ebp pilus genes** | **Ref** |
| --- | --- | --- | --- | --- | --- |
| 1 | SJH-737 | *Enterococcus* unclassified VVC95 1-77 Urine | UTI isolate | Yes | This study |
| 2 | SJH-742 | *Enterococcus* unclassified FDS124 15-682 Urine | UTI isolate | Yes | This study |
| 3 | SJH-749 | *Enterococcus* unclassified PM21307 | UTI isolate | Yes | This study |
| 4 | SJH-1220 | *Enterococcus faecalis* OG1X | Laboratory strain | Yes | (*1, 2*) |
| 5 | SJH-1221 | *Enterococcus faecalis* OG1SS pCF10 | Laboratory strain | Yes | (*3*) |
| 6 | SJH-1224 | *Enterococcus faecalis* FA2-2 | Laboratory strain | Yes | (*4*) |
| 7 | SJH-1225 | *Enterococcus faecalis* FA2-2 pAD1 | Laboratory strain | Yes | (*4*) |
| 8 | SJH-1226 | *Enterococcus faecalis* FA2-2 pAM944 | Laboratory strain | Yes | (*5*) |
| 9 | SJH-1227 | *Enterococcus faecalis* FA2-2 pAM947 | Laboratory strain | Yes | (*5*) |
| 10 | SJH-1228 | *Enterococcus faecalis* FA2-2 pAM9058 | Laboratory strain | Yes | (*5*) |
| 11 | SJH-1309 | Enterococcus faecalis MMH594 | Bacteremia isolate | Yes | (*6*) |
| 12 | SJH-1310 | Enterococcus faecalis MMH594b | Bacteremia isolate | Yes | (*6*) |
| 13 | SJH-1311 | *Enterococcus faecalis* HG101 | Laboratory strain | Yes | (*7*) |
| 14 | SJH-1312 | *Enterococcus faecalis* TX5266 | Laboratory strain | Yes | (*8, 9*) |
| 15 | SJH-1317 | *Enterococcus faecalis* UTI EF2340 | UTI isolate | Yes | This study |
| 16 | SJH-1318 | *Enterococcus faecalis* UTI EF5245 | UTI isolate | Yes | This study |
| 17 | SJH-1319 | *Enterococcus faecalis* UTI EF2116 | UTI isolate | Yes | This study |
| 18 | SJH-1320 | Enterococcus faecalis VRE B 1-22 | Blood isolate | Yes | This study |
| 19 | SJH-1321 | *Enterococcus faecalis* VRE B 2-22 | Blood isolate | Yes | This study |
| 20 | SJH-1322 | *Enterococcus faecalis* VRE B 3-22 | Blood isolate | Yes | This study |
| 21 | SJH-1323 | *Enterococcus faecalis* | Clinical isolate | Yes | This study |
| 22 | SJH-1324 | *Enterococcus faecalis* VRE B 5-22 | Blood isolate | Yes | This study |
| 23 | SJH-1325 | *Enterococcus faecalis* VRE B 6-22 | Blood isolate | Yes | This study |
| 24 | SJH-1326 | *Enterococcus faecalis* VRE B 7-22 | Blood isolate | Yes | This study |
| 25 | SJH-1327 | *Enterococcus faecalis* VRE B 8-22 | Blood isolate | Yes | This study |
| 26 | SJH-1328 | *Enterococcus faecalis* VRE B 9-22 | Blood isolate | Yes | This study |
| 27 | SJH-1329 | *Enterococcus faecalis* VRE B 10-22 | Blood isolate | Yes | This study |
| 28 | SJH-1330 | *Enterococcus faecalis* VRE B 11-22 | Blood isolate | Yes | This study |
| 29 | SJH-1331 | *Enterococcus faeccium* H Ch Gut C5 | Gut isolate | Yes | This study |
| 30 | SJH-1332 | *Enterococcus faeccium* H Ch Gut C4 | Gut isolate | Yes | This study |
| 31 | SJH-1333 | *Enterococcus faeccium* H Ch Gut A4 | Gut isolate | Yes | This study |
| 32 | SJH-1334 | *Enterococcus faeccium* H Ch Gut B8 | Gut isolate | Yes | This study |
| 33 | SJH-1335 | *Enterococcus faeccium* H Ch Gut A3 | Gut isolate | Yes | This study |
| 34 | SJH-1336 | *Enterococcus faeccium* H Ch Gut H8 | Gut isolate | Yes | This study |
| 35 | SJH-1337 | *Enterococcus faecalis* H Ch Gut A2 | Gut isolate | Yes | This study |
| 36 | SJH-1338 | *Enterococcus faecalis* H Ch Gut E2 | Gut isolate | Yes | This study |
| 37 | SJH-1339 | *Enterococcus faecalis* H Ch Gut D2-2 | Gut isolate | Yes | This study |
| 38 | SJH-1340 | *Enterococcus faeccium* H Ch Gut C3 | Gut isolate | Yes | This study |
| 39 | SJH-1341 | *Enterococcus faecalis* H Ch Gut E9-2 | Gut isolate | Yes | This study |
| 40 | SJH-1350 | *Enterococcus faecalis* V583 | Blood isolate | Yes | (*10*) |
| 41 | SJH-1357 | *Enterococcus faecalis* OG1X D0402 | Laboratory strain | Yes | This study |
| 42 | SJH-1365 | *Enterococcus* unclassified FOS124 | Clinical isolate | Yes | This study |
| 43 | SJH-1379 | *Enterococcus* unclassified VRE 05-2 | Clinical isolate | Yes | This study |
| 44 | SJH-1380 | *Enterococcus* unclassified VRE 05-3 | Clinical isolate | Yes | This study |
| 45 | SJH-1381 | *Enterococcus* unclassified VRE 05-4 | Clinical isolate | Yes | This study |
| 46 | SJH-1382 | *Enterococcus* unclassified VRE 05-6 | Clinical isolate | Yes | This study |
| 47 | SJH-1383 | *Enterococcus* unclassified VRE 05-7 | Clinical isolate | Yes | This study |
| 48 | SJH-1384 | *Enterococcus* unclassified VRE 05-8 | Clinical isolate | Yes | This study |
| 49 | SJH-1385 | *Enterococcus* unclassified VRE 05-11 | Clinical isolate | Yes | This study |
| 50 | SJH-1386 | *Enterococcus* unclassified VRE 05-12 | Clinical isolate | Yes | This study |
| 51 | SJH-1387 | *Enterococcus* unclassified VRE 05-25 | Clinical isolate | Yes | This study |
| 52 | SJH-1388 | *Enterococcus* unclassified VRE 05-28 | Clinical isolate | Yes | This study |
| 53 | SJH-1417 | *Enterococcus faecalis* OG1X DMprF | Laboratory strain | Yes | This study |
| 54 | SJH-2136 | *Enterococcus gallinarum* | Clinical isolate | Yes | This study |
|  | SJH-1994 | OG1RF (Rif/Fus) | EbpA^+^, EbpB^+^, EbpC^+^, SrtC^+^, SrtA^+^ | Yes | (*11*) |
| **OG1RF chromosomal deletion mutants** | SJH-1995 | *ΔebpABCΔsrtC* (Rif/Fus) | EbpA^-^, EbpB^-^, EbpC^-^, SrtC^-^, SrtA^+^ | No | (*12*) |
|  | SJH-1996 | Δ*ebpA*(Rif/Fus) | EbpA^-^, EbpB^+^, EbpC^+^, SrtC^+^, SrtA^+^ | Partial | (*12*) |
|  | SJH-2001 | *ebpA*^AWAGA^ (Rif/Fus) | EbpA^D315A, S317A, S319A^, EbpB^+^, EbpC^+^, SrtC^+^, SrtA^+^ | EbpA point mutations | (*12*) |

1. Y. Ike, D. B. Clewell, R. A. Segarra, M. S. Gilmore, Genetic analysis of the pAD1 hemolysin/bacteriocin determinant in Enterococcus faecalis: Tn917 insertional mutagenesis and cloning. *Journal of bacteriology* **172**, 155-163 (1990); published online EpubJan (

2. Y. Ike, R. A. Craig, B. A. White, Y. Yagi, D. B. Clewell, Modification of Streptococcus faecalis sex pheromones after acquisition of plasmid DNA. *Proceedings of the National Academy of Sciences of the United States of America* **80**, 5369-5373 (1983); published online EpubSep (

3. P. J. Christie, G. M. Dunny, Identification of regions of the Streptococcus faecalis plasmid pCF-10 that encode antibiotic resistance and pheromone response functions. *Plasmid* **15**, 230-241 (1986); published online EpubMay (

4. D. B. Clewell, P. K. Tomich, M. C. Gawron-Burke, A. E. Franke, Y. Yagi, F. Y. An, Mapping of Streptococcus faecalis plasmids pAD1 and pAD2 and studies relating to transposition of Tn917. *Journal of bacteriology* **152**, 1220-1230 (1982); published online EpubDec (

5. J. W. Chow, L. A. Thal, M. B. Perri, J. A. Vazquez, S. M. Donabedian, D. B. Clewell, M. J. Zervos, Plasmid-associated hemolysin and aggregation substance production contribute to virulence in experimental enterococcal endocarditis. *Antimicrobial agents and chemotherapy* **37**, 2474-2477 (1993); published online EpubNov (

6. N. Shankar, C. V. Lockatell, A. S. Baghdayan, C. Drachenberg, M. S. Gilmore, D. E. Johnson, Role of Enterococcus faecalis surface protein Esp in the pathogenesis of ascending urinary tract infection. *Infection and immunity* **69**, 4366-4372 (2001); published online EpubJul (10.1128/IAI.69.7.4366-4372.2001).

7. L. E. Hancock, M. S. Gilmore, The capsular polysaccharide of Enterococcus faecalis and its relationship to other polysaccharides in the cell wall. *Proceedings of the National Academy of Sciences of the United States of America* **99**, 1574-1579 (2002); published online EpubFeb 5 (10.1073/pnas.032448299).

8. X. Qin, K. V. Singh, G. M. Weinstock, B. E. Murray, Characterization of fsr, a regulator controlling expression of gelatinase and serine protease in Enterococcus faecalis OG1RF. *Journal of bacteriology* **183**, 3372-3382 (2001); published online EpubJun (10.1128/JB.183.11.3372-3382.2001).

9. Y. A. Su, M. C. Sulavik, P. He, K. K. Makinen, P. L. Makinen, S. Fiedler, R. Wirth, D. B. Clewell, Nucleotide sequence of the gelatinase gene (gelE) from Enterococcus faecalis subsp. liquefaciens. *Infection and immunity* **59**, 415-420 (1991); published online EpubJan (

10. D. F. Sahm, J. Kissinger, M. S. Gilmore, P. R. Murray, R. Mulder, J. Solliday, B. Clarke, In vitro susceptibility studies of vancomycin-resistant Enterococcus faecalis. *Antimicrobial agents and chemotherapy* **33**, 1588-1591 (1989); published online EpubSep (

11. G. M. Dunny, B. L. Brown, D. B. Clewell, Induced cell aggregation and mating in Streptococcus faecalis: evidence for a bacterial sex pheromone. *Proceedings of the National Academy of Sciences of the United States of America* **75**, 3479-3483 (1978); published online EpubJul (

12. H. V. Nielsen, P. S. Guiton, K. A. Kline, G. C. Port, J. S. Pinkner, F. Neiers, S. Normark, B. Henriques-Normark, M. G. Caparon, S. J. Hultgren, The metal ion-dependent adhesion site motif of the Enterococcus faecalis EbpA pilin mediates pilus function in catheter-associated urinary tract infection. *mBio* **3**, e00177-00112 (2012)10.1128/mBio.00177-12).
